# Differential Precipitation of Mg(OH)_2_ from CaSO_4_·2H_2_O Using Citrate as Inhibitor—A Promising Concept for Reagent Recovery from MgSO_4_ Waste Streams

**DOI:** 10.3390/molecules25215012

**Published:** 2020-10-29

**Authors:** Szilveszter Ziegenheim, Márton Szabados, Zoltán Kónya, Ákos Kukovecz, István Pálinkó, Pál Sipos

**Affiliations:** 1 Department of Inorganic and Analytical Chemistry, University of Szeged, Dóm tér 7, H-6720 Szeged, Hungary; ziegenheimsz@chem.u-szeged.hu; 2 Material and Solution Structure Research Group, Institute of Chemistry, University of Szeged, Aradi Vértanúk tere 1, H-6720 Szeged, Hungary; szabados.marton@chem.u-szeged.hu (M.S.); palinko@chem.u-szeged.hu (I.P.); 3 Department of Organic Chemistry, University of Szeged, Dóm tér 8, H-6720 Szeged, Hungary; 4 Department of Applied and Environmental Chemistry, University of Szeged, Rerrich Béla tér 1, H-6720 Szeged, Hungary; konya@chem.u-szeged.hu (Z.K.); kakos@chem.u-szeged.hu (Á.K.); 5 MTA-SZTE Reaction Kinetics and Surface Chemistry Research Group, Rerrich B. tér 1, H-6720 Szeged, Hungary

**Keywords:** gypsum, magnesium hydroxide, inhibition, citric acid, precipitation separation

## Abstract

In hydrometallurgical processing and acidic wastewater treatment, one of the neutralizing agents employed is MgO or Mg(OH)_2_. At the end of this process, the resulting solution, which is rich in SO_4_^2−^ and Mg^2+^ is treated with lime to remove (or minimize the amount) of these ions via the precipitation of Mg(OH)_2_ and CaSO_4_·2H_2_O (gypsum). In our work, an attempt was made to separate the two solids by increasing the induction time of the gypsum precipitation, thus regenerating relatively pure Mg(OH)_2_ which could be reused in wastewater treatments or hydrometallurgical processing circuits, and in this way, significantly enhancing the economic viability of the process. During our experiments, the reaction of an MgSO_4_ solution with milk of lime prepared from quicklime was studied. The effects of a range of organic additives, which can slow down the precipitation of gypsum have been assessed. The process was optimized for the most promising inhibiting agent—that is, the citrate ion. The reactions were continuously monitored in situ by conductometric measurements with parallel monitoring of solution pH and temperature. ICP-OES measurements were also carried out on samples taken from the reaction slurry. The composition of the precipitating solids at different reaction times was established by powder XRD and their morphology by SEM. Finally, experiments were carried out to locate the additive after the completion of the precipitation reaction to get information about its potential reuse.

## 1. Introduction

Solid precipitation from supersaturated solutions is a common phenomenon; however, in industrial processes it can cause several nuisances—e.g., via scaling or as a co-precipitating side product. Scaling can reduce the efficiency of heat-exchanging surfaces (water cooling systems), clog membranes and tubes (reverse osmosis units, water injection systems in oil and gas production), while co-precipitating solids are present as impurities in the final products. One of the most commonly precipitating solids is CaSO_4_·2H_2_O, gypsum; therefore, the detailed understanding of its crystallization is necessary in a variety of processes, which are adversely affected by its precipitation.

**Scheme 1:**
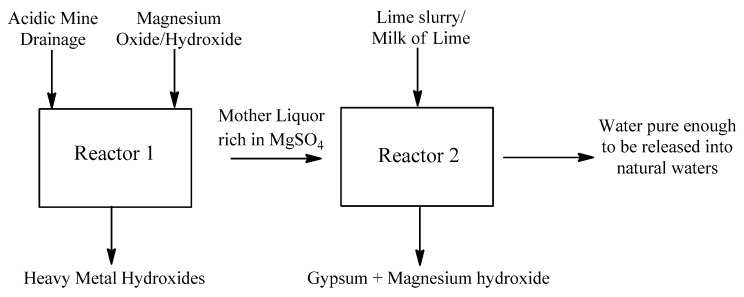
Two-step neutralization of acidic mine drainage using magnesium oxide/hydroxide for separate precipitation of metal hydroxides.

An important field where gypsum is present in high quantities is acidic wastewater treatment. Normally, these acidic waters contain heavy metal and sulfate ions, which are precipitated as metal hydroxides and calcium sulfate using lime for neutralization [1]. This way, the remaining water can be released to natural waters. However, the sludge, which forms, requires further treatment and must be disposed. Using magnesium oxide/hydroxide as the first step of a two-step neutralization process can provide an alternative, and this way, gypsum can be precipitated separately from the heavy metal hydroxides [2,3], as shown in Scheme 1. The process could also be useful in the desalination process [4].

In this work, an attempt is made to improve the latter method further by separating the precipitation of magnesium hydroxide and gypsum in solution by using additives. This has the potential of enhancing the economic and environmental viability of the process, and the results can be extended to separate gypsum precipitation in time from rapidly precipitating solids other than Mg(OH)_2_.

The solubility of Mg(OH)_2_ (K_sp_ is around 1.38 × 10^−11^) is significantly lower than that of CaSO_4_ (K_sp_ is around 2.5 × 10^−5^) [5,6]. The nucleation of Mg(OH)_2_ is also highly dependent on the ion concentrations, and is very fast (practically instantaneous) at high supersaturation [7,8,9,10]. The inhibition of the crystallization of Mg(OH)_2_ was found to be rather difficult when conventional additives were used [10,11,12,13].

The kinetics of gypsum precipitation, which is significantly slower than that of Mg(OH)_2_, was investigated in several studies [6,14,15,16,17,18]. As it is a common scaling and precipitating solid, the inhibition of its crystallization has been the focus of various studies since the 1950s [19]. The effect of various additives has been scrutinized over recent decades. Various metal ions were found to influence the precipitation of gypsum, affecting both crystal growth and morphology [20,21,22,23], even in the presence of certain amino acids [24]. Organic phosphonates were also demonstrated to be efficient inhibitors in gypsum precipitation [25,26,27,28]. Polymeric compounds with carboxylic groups also proved to be effective in the inhibition of gypsum precipitation—the most popular is perhaps polyacrylic acid; however, some other “green” alternatives were also efficient [29,30,31,32]. Looking into small-molecule simple carboxylic acids, the performance of citric acid seemed to be remarkable [33] and accordingly its inhibiting effects were investigated in more detail [34,35,36].

It seems plausible that the inhibition of the precipitation of gypsum is much more promising than that of magnesium hydroxide. Accordingly, scouting experiments were performed in a stoichiometric Ca^2+^ + SO_4_^2−^ reaction at pH ≈ 7 using a number of additives (for details, see Supplementary Materials, Appendix A. In this paper, the optimization of the reaction conditions to separate the precipitated magnesium hydroxide from gypsum in time using one particular inhibitor from the aforementioned additives—citrate ions—is presented.

## 2. Results

### 2.1. Effect of the Reaction Conditions on the Kinetics of the MgSO_4_ + Ca(OH)_2_ + 2H_2_O → Mg(OH)_2_ + CaSO_4_·2H_2_O Reaction

**Figure 1:**
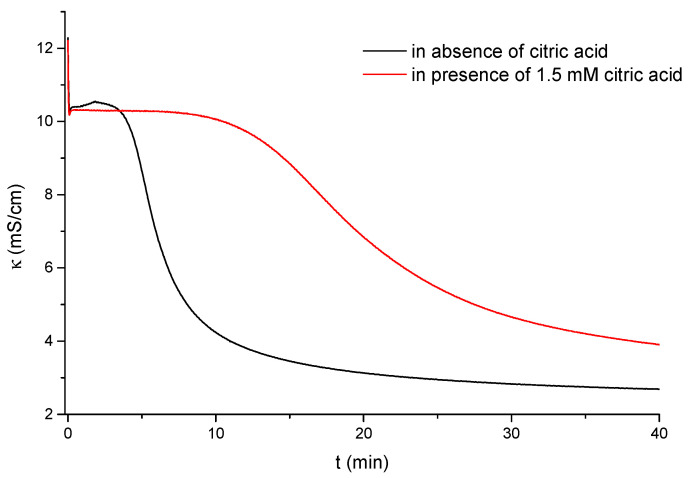
Variation of solution conductivity during the reaction of MgSO_4_ + Ca(OH)_2_ + 2 H_2_O → Mg(OH)_2_ + CaSO_4_·2H_2_O with identical (0.1 M) initial reactant concentrations in the absence and in the presence of 1.5-mM citric acid (T = 20 °C; in distilled water media without any supporting electrolyte).

During our experiments, the stoichiometric reaction of MgSO_4_ + Ca(OH)_2_ + 2 H_2_O → Mg(OH)_2_ + CaSO_4_·2H_2_O was studied using milk of lime (MoL)—prepared from quicklime—as a Ca(OH)_2_ source. The effects of the reaction conditions and added citric acid—as an inhibitor for gypsum precipitation—were investigated. The reactions were monitored in situ with conductometry and the pH and temperature of the reaction mixture were also recorded. The pH of the initial MgSO_4_ solution was found to be neutral without the additive, but it dropped to around pH ≈ 3 upon citric acid addition; however, it raised to pH ≈ 11.1–11.2 when MoL was added to the mixture, which, according to the literature, coincides with the nucleation pH of Mg(OH)_2_ [7]. The variation of solution conductivity vs. time during the reactions is shown in Figure 1.

Upon adding MoL to the aqueous solution of MgSO_4_, a sharp drop in the conductivity of the reaction mixture instantaneously appeared, both in the presence and absence of the inhibitor. This drop is most probably the result of two effects—the formation of the Mg(OH)_2_ precipitate and, parallel to this, the dissolution of Ca(OH)_2_. Then a plateau can be observed in both systems, corresponding to the induction period prior to gypsum precipitation. The smooth decrease in the conductivity corresponds to the precipitation of CaSO_4_·2H_2_O. It can be seen that the addition of citric acid significantly increased the induction period of gypsum precipitation, under the specific conditions shown above, from around 3 to 10 min.

The induction times of the reactions were estimated using the data of the conductivity measurements. The induction time was estimated as the time required for the conductivity of the slurry to drop by 0.2 mS/cm, which is twice the standard error of the measuring method. In addition, to quantify the reaction rate more exactly and comparably, the measured conductivity data were fitted with using the following equation:

$$y=b+\frac{a-b}{1+{\left(\frac{t}{i}\right)}^{c}}$$

where *a* is the initial (after the first drop) and *b* is the final conductivity of the reaction mixture, *t* is the reaction time, *c* is the slope factor and *i* is the half-reaction time (time needed for half of the reactants to react). During the fittings, the initial and final conductivities were held constant at the experimental values. With this half-reaction time the reaction rates are quantitatively comparable.

**Figure 2:**
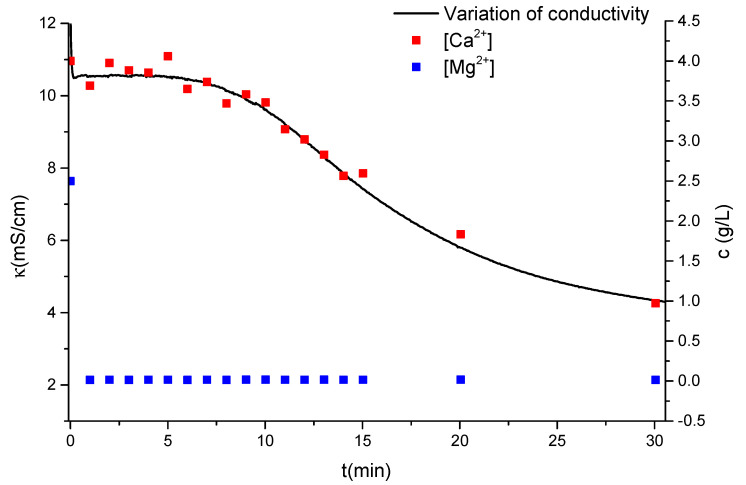
Comparison of the results of in situ conductivity measurement (left Y axis) and ICP-OES measurements (right Y axis); latter were performed on samples of filtered mother liquors taken during the stoichiometric reaction of MgSO_4_ + Ca(OH)_2_ → Mg(OH)_2_ + CaSO_4_·2H_2_O with identical (0.1 M) initial reactant concentrations, containing 1.5-mM citric acid inhibitor in distilled water (initial ionic concentrations were calculated from the weight of the added salts; T = 20 °C; in distilled water media without any supporting electrolyte).

To confirm the above interpretation of the conductivity vs. time profiles shown in Figure 1, the concentrations of Ca^2+^ and Mg^2+^ during the experiment with solutions containing the inhibitor were determined. Samples were taken intermittently at given times from the reaction slurry. After quick filtration (taking approximately 5–10 s with a syringe filter) and hundred-fold dilution, ICP-OES measurements yielded the Mg^2+^ and Ca^2+^ concentration of the solution (Figure 2).

The results seem to verify that the magnesium content of the solution practically instantly precipitates from the slurry as Mg(OH)_2_ (the ICP-OES measurements yielded Mg^2+^ concentrations in the range of 0.3–0.6% of the initial Mg^2+^ concentration). The Ca^2+^ and SO_4_^2^^−^ ions remain in solution for a significantly longer period of time before gypsum precipitation commences. It can also be seen that the Ca^2+^ variations of the solution concentrations obtained from ICP-OES are superimposable with the conductometric data, confirming the viability of conductometry as in situ means for monitoring the precipitation of gypsum in this system.

**Figure 3:**
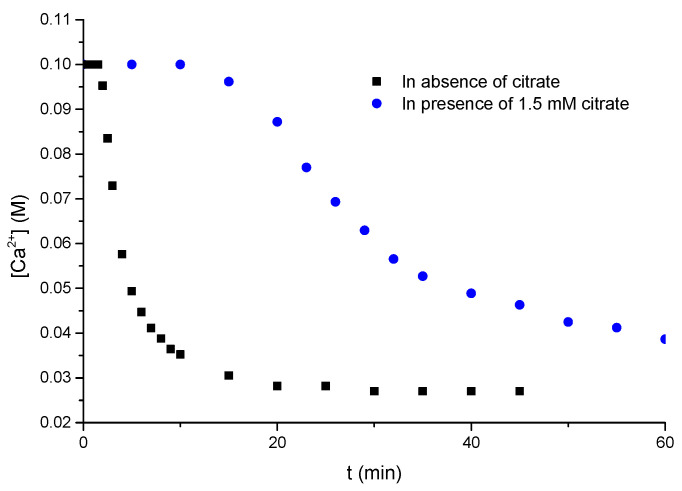
The variation of [Ca^2+^] during the reaction of Na_2_SO_4_ + CaCl_2_ + 2 H_2_O → 2 NaCl + CaSO_4_·2H_2_O with 0.1-M initial reactant concentration in absence and in presence of 1.5-mM citrate.

To get a more detailed picture on the inhibition effect of citric acid, its effect on the precipitation of gypsum was also studied in the reaction of Na_2_SO_4_ + CaCl_2_ + 2 H_2_O → 2 NaCl + CaSO_4_·2H_2_O with a 0.1 M initial reactant concentration, thus excluding the effect of Mg(OH)_2_ present in the target system. In these reactions, trisodium citrate was added as an inhibitor, to avoid the decrease in the pH and to have the inhibitor in the same form as in the target reaction. These reactions were thermostated at 25 °C and carried out in a smooth-surface cylindrical PTFE vessel to avoid any wall effect. The variation of the [Ca^2+^] was calculated from the conductometric measurements using two-point calibration using the initial and final [Ca^2+^] concentration. The results are shown in Figure 3.

It can be seen that the citrate significantly retards the crystallization of gypsum. The half-reaction time in absence of citrate was approximately 3.5 min which increased to 28 min in the presence of 1.5-mM citrate inhibitor. Under these circumstances, the inhibition seems to be more efficient than in the target reaction under identical conditions, which suggests some interaction between the Mg(OH)_2_ and the additive.

**Figure 4:**
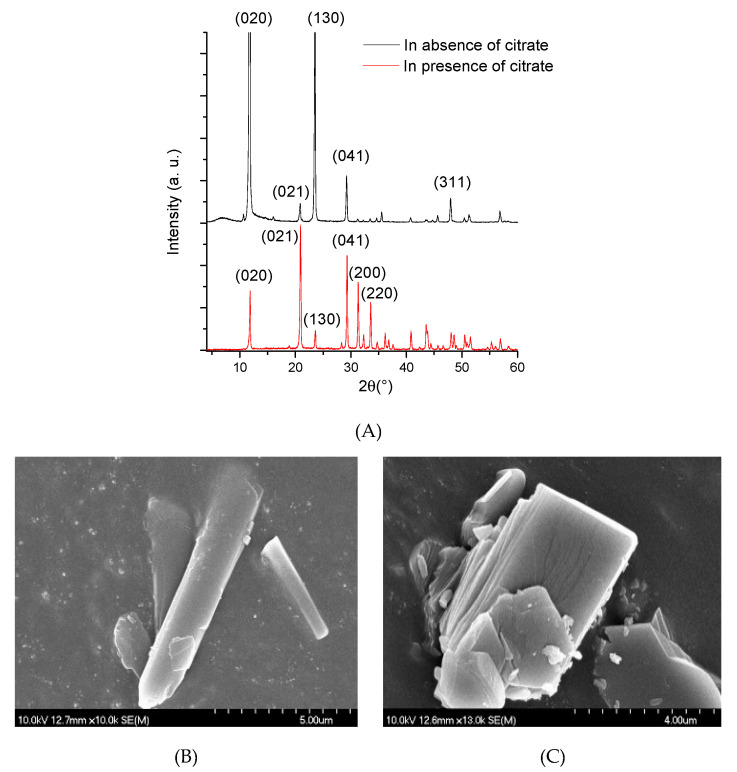
Structural and morphological investigation of gypsum precipitated during the reaction of Na_2_SO_4_ + CaCl_2_ + 2 H_2_O → 2 NaCl + CaSO_4_·2H_2_O. (**A**): XRD patterns of gypsum precipitated in the absence and in the presence of citrate (main miller indices were identified using the JCPDS database—CaSO_4_·2H_2_O: # 21-0816). Bottom: SEM images of gypsum precipitated in absence (**B**) and in presence (**C**) of citrate.

The solids precipitating in the model reactions were studied by using X-ray diffractometry and scanning electron microscopy. The results are shown in Figure 4.

From the diffractograms, the only detectable crystalline solid is gypsum; however, the intensity of the reflections and their ratio to each other change immensely between the ones precipitated in absence or in the presence of citrate. This implies that the morphology of the two solids is significantly different. SEM images support this assumption. While in the absence of citrate, the gypsum only precipitates into well-shaped, rod-like crystals; in the solids crystallized in the presence of citrate, only plates can be found. Morphological differences analogous to these were already reported in the literature by others [33,34,35,36,37,38,39].

The inhibition effect observed—i.e., the increase in the induction time—may be partly due to Ca^2+^ complexation by citrate in solution (decreasing the degree of supersaturation) [37]. This is, however, likely to be a minor effect due to the low concentration of the inhibitor relative to that of the Ca^2+^ and SO_4_^2^^−^ ions. The surface adsorption, which has been evidenced for citrate as well as for other organic compounds with carboxylate functionalities [33,35,38], is indeed more relevant here. It was demonstrated [33,38] that citrate adsorbs onto the active growth faces of gypsum. This way, it inhibits the growth in these directions and results in morphological changes in the forming crystals. In the absence of citrate, the (uninhibited) growth along the c axis results in the formation of typical, long, needle-like crystals (Figure 4B) [39]. This is inhibited in the presence of citrate, and crystal growth is favored in the a and b directions, resulting in the formation of plate-like crystals. Badens et al. [33] suggested that for efficient adsorption (or surface complexation) of citrate, the distance between the surface calcium ions and that of the two oxygen atoms on the two carboxylic groups has to match; for this, the faces (1 2 0) and (1 1 1) appeared to be the optimal ones [33,39]. In a recent work, the surface complexation of a citrate monolayer was demonstrated by using a variety of experimental means and corroborated by MD simulations [36]. In this work [36], the difference between the inhibiting efficiency of citrate and tartrate [33] was also possible to be explained.

**Figure 5:**
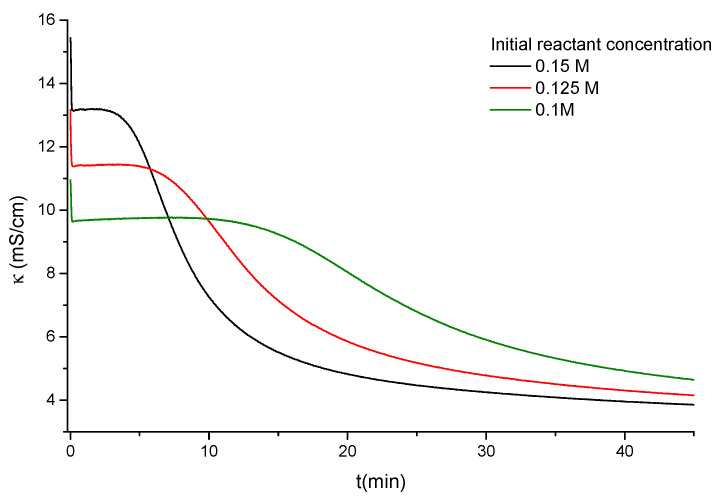
Effect of increasing reactant concentration on the conductivity vs time profiles of the reaction of MgSO_4_ + Ca(OH)_2_ + 2 H_2_O → Mg(OH)_2_ + CaSO_4_·2H_2_O. During the reactions, the additive-to-reactant ratio was held constant (the molar ratio of Ca:Mg:citric acid = 0.1:0.1:0.0015), the initial reactant concentrations are shown in the figure (T = 20 °C; in distilled water media without any supporting electrolyte).

To investigate the kinetics of the reaction of MgSO_4_ + Ca(OH)_2_ + 2 H_2_O → Mg(OH)_2_ + CaSO_4_·2H_2_O further, the initial concentrations were systematically increased, while the reactant-to-additive ratio was kept constant. The results of these measurements are shown in Figure 5.

It can be seen that even as the inhibitor concentration was increased proportionally to the increased reactant concentrations, the rate of the gypsum precipitation reaction increased significantly: the half-reaction time of the reaction with 0.15-M initial reactant concentrations was 8.5 min while with 0.1 M it was found to be 22.8 min. This phenomenon is somewhat expected, as the high agitation speed, the increasing supersaturation ratio and the bigger amount of solid (and thus potential nucleation sites) present in the reaction mixture all facilitate the precipitation of gypsum. This may be undesirable in practical applications, and must be somehow taken into account during the secondary step of acidic mine drainage treatment, as the MgSO_4_-rich solutions coming from the first step may vary in concentration. A potential solution for this can be dilution or increase the additive used. If it can be carried out economically, the latter is probably favored, because it does not make it necessary to increase the amount of liquid to be treated.

**Figure 6:**
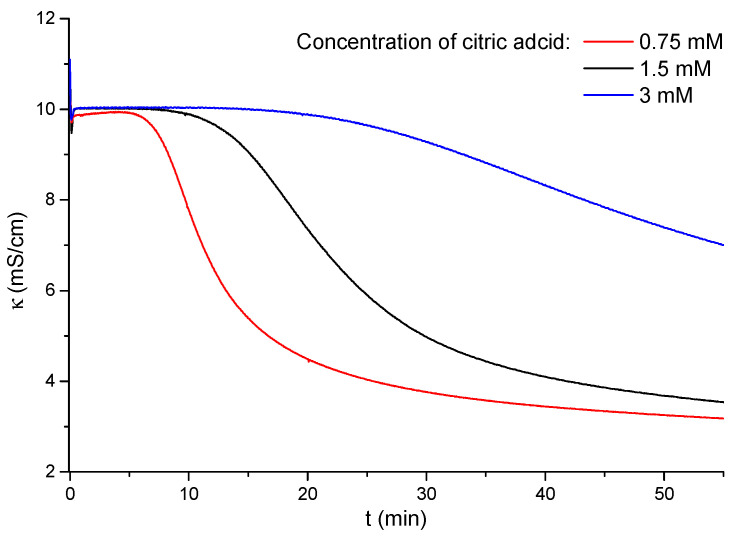
Variation of conductivity in the reaction of MgSO_4_ + Ca(OH)_2_ + 2 H_2_O → Mg(OH)_2_ + CaSO_4_·2H_2_O with 0.1-M initial reactant concentration, using different amounts of citric acid as inhibitor (T = 20 °C; in distilled water media without any supporting electrolyte).

To study the effect of the variation of additive dosage, the reaction with 0.1 M initial reactant concentrations was carried out, varying the amount of citrate added (Figure 6).

The added amount of citric acid exerts a major effect on the rate of gypsum precipitation. When the amount used previously (1.5 mM) was doubled, the half-reaction time was also almost doubled: with 3 mM it was 56 min compared to 23 min with 1.5 mM. As the inhibiting effect is increased significantly this could provide a viable method to compensate the effect of increasing reactant concentrations. However, increasing the amount of additive can lower the cost-efficiency of the process, and while citric acid is a “green” additive as it is commonly present in nature, it still cannot be used in large amounts. To overcome these challenges, its potential to be reused without adding a new, costly step to the process must be investigated—this will be discussed later in this work (see Section 2.3).

During our experiments, the temperature of the reaction mixtures was always monitored. It was observed that the initial temperature of the MgSO_4_ solution did not change significantly upon adding the MoL to it, presumably because the various heat effects roughly cancelled each other out. It was also observed that the temperature of the reaction mixture remained constant during the precipitation of the gypsum too—within ±0.5 °C. Accordingly, the test reactions always commenced at the actual room temperature and remained approximately constant during an experimental run. After several repeated test reactions, it was observed that the temperature of the reaction mixture also affects the rate of reaction. The extent of this is qualitatively in accord with the Arrhenius law. The correlation with the rate of the reaction is summed up in Table 1.

As it can be seen, even relatively small temperature changes result in significant variations in the reaction rate. At a lower temperature the reaction was slower, and it seems natural that the reaction becomes faster with increasing temperature—the temperature dependence of the solubility of gypsum can also play a role in this [40].

Since the temperature can also influence the reaction significantly, it can be seen that the details of the experimental procedure must be considered as a whole to suggest an operational setup for the separation of magnesium hydroxide and gypsum, and these conditions must be considered specifically for the given site, where it is used.

It is also worth mentioning that during the treatment of wastewaters from acidic mine drainages the neutralized fluids are likely to contain a range of impurities, such as other metal ions as well as some organic components. Most of the metal ions should be precipitated in the first step of neutralization with magnesium oxide/hydroxides in their hydroxide form; however, some studies suggest that they can influence the precipitation of gypsum even in trace amounts [20,22,23]. However, as the pH during the second step can increase up to ~11.2, and it is regulated by the reactants and products, the other residual metal ions are most likely precipitated as hydroxides at this stage.

A more relevant factor for modifying the kinetics of the reaction in industrial systems can be assigned to organic impurities. While in this work the solutions were made up using distilled water, the Ca(OH)_2_ source was made of natural limestone, therefore probably containing some organic impurities. According to the literature and our previous works, additives containing carboxylic acid and phosphonate functional groups can effectively inhibit the precipitation of gypsum [25,26,27,28,29,30,31,32,33,34,35,36,37,38,39], these types of impurities may, however, even improve the inhibition effect. On the other hand, compounds reacting with these organic molecules or interacting with the precipitants can be responsible for a variety of complications. Therefore, the effects associated with the auxiliary components of the starting solution must always be tested to fit the conditions of the process to the specific requirements.

### 2.2. Analysis of the Precipitating Solids and the Effect of Washing

**Scheme 2:**
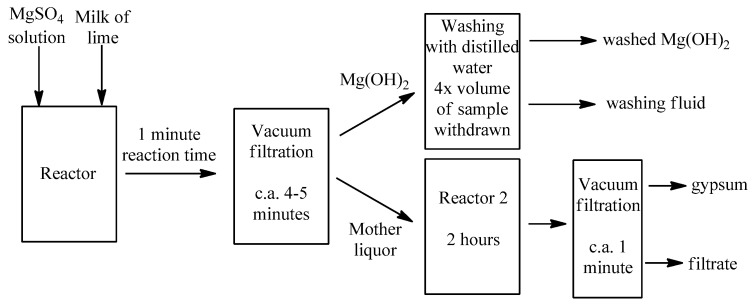
Experimental procedure for separating the precipitating solids during the MgSO_4_ + Ca(OH)_2_ + 2 H_2_O → Mg(OH)_2_ + CaSO_4_·2H_2_O reaction.

To characterize the solids precipitating from the reaction mixture (reaction conditions as shown in Figure 1), samples were taken from the reaction slurry. To separate the two precipitates, a portion of the slurry was withdrawn at a 1 min reaction time. After a relatively quick filtration (taking ca. 5 min) the separated mother liquor, which is heavily supersaturated with respect to gypsum, was agitated further for 2 h to precipitate the excess calcium sulfate in the solution. The solid filtered first mainly contained magnesium hydroxide; however, even with quick filtration, after drying, there was always some gypsum remaining in the solids. The reason for this is probably the remaining mother liquor on the surface of the solid, which still contains gypsum, which precipitates during the various manipulations and the subsequent drying. To eliminate these impurities, the samples were washed. After washing with different amounts of distilled water, we found that the optimal volume is 3–4 times the volume of the withdrawn sample. The separation process is presented in Scheme 2.

**Figure 7:**
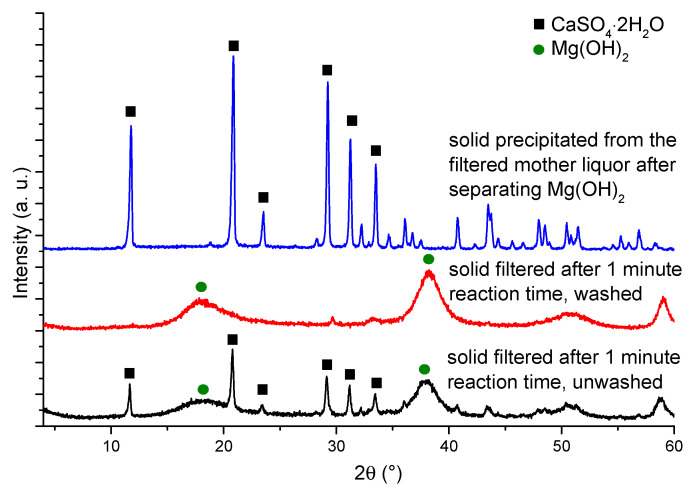
XRD patterns of the separated, precipitated solids; main reflections were identified using the JCPDS database (CaSO_4_·2H_2_O: #21-0816 and Mg(OH)_2_: #84-2164) Washing of Mg(OH)_2_ with distilled water, four times the volume of taken sample.

To enhance the efficiency of the process the properties of the products were investigated. First, the solids were analyzed with powder XRD; the results are shown in Figure 7.

Without sufficient aging, Mg(OH)_2_ tends to precipitate in poorly defined, most often amorphous crystals. The wide reflections appearing on the diffractograms suggest that this happens during the reactions in our hands, as the residence time is not sufficient for the formation of well-defined Mg(OH)_2_ crystals. On the other hand, the reflections corresponding to gypsum were found always to be intensive and sharp making it easy to identify even when present in relatively small amounts. It can be seen that the washing of the initially filtered solid resulted in pure Mg(OH)_2_, and no typical reflection of gypsum is seen on the diffractogram.

Since washing seemed to be necessary to achieve a reasonable purity of Mg(OH)_2_, the effect of washing was studied with systems at various initial concentrations. The optimal time-window of initial filtration was also tested by withdrawing samples from the reaction slurry at given times. The washing procedure was mentioned before—it was carried out with distilled water, four times the volume of the taken sample. The results are summed up in Table 2.

The time-window, where one can still wash out the gypsum from the Mg(OH)_2_, decreases quickly with an increasing initial reactant concentration. Besides the faster reaction rate, the bigger amounts of solids in the slurry also present a challenge by making the filtration process longer resulting in more gypsum precipitated.

**Figure 8:**
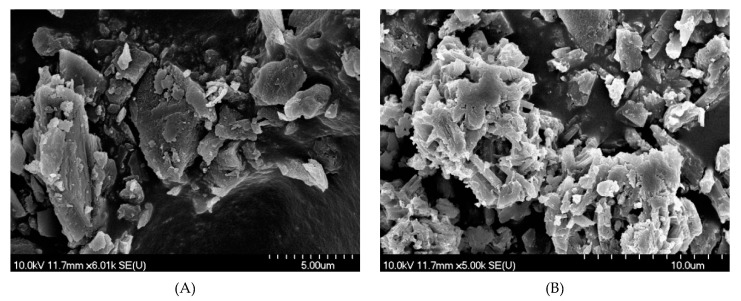
SEM images of the precipitated Mg(OH)_2_ (**A**): washed sample (does not contain gypsum according to XRD) and (**B**): unwashed sample (some gypsum present according to XRD).

To look into the properties of the solids in more detail, SEM images of the washed and unwashed Mg(OH)_2_ samples were recorded. The pictures are presented in Figure 8.

The SEM images of the precipitated magnesium hydroxide attest that the solids did not form regular crystals and that they have no clear morphology. When looking at the samples containing some gypsum impurities, no observable morphology can be seen; however, the small bumps and spikes hint that gypsum precipitated on the surface and edges of the Mg(OH)_2_.

**Figure 9:**
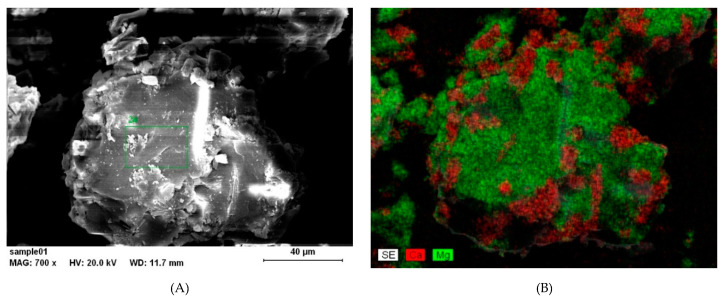
SEM-EDX picture (**A**) and elemental map (**B**) of a representative unwashed Mg(OH)_2_ precipitate.

To investigate this phenomenon further and back up our earlier results, SEM-EDX measurements were carried out on the same solid samples. Looking at the washed Mg(OH)_2_ samples, we found minute amounts of calcium in the solids (the Ca concentration was estimated to be less than 1 at%). The gypsum content of the unwashed samples was found to be much higher, and the elemental maps of these samples revealed the distribution of calcium and magnesium in the solids (Figure 9). The elemental map of the unwashed Mg(OH)_2_, which precipitated proves that magnesium and calcium are located separately, and the gypsum precipitates on the surface of the Mg(OH)_2_ crystals present.

### 2.3. The Location of Citrate Ions after the Completion of both Precipitation Reactions

Determining the destination of the added citric acid is necessary both from an economic and environmental point of view. To determine where the additive ends up, initially, UV-spectrophotometric measurements were carried out on the mother liquor obtained after the precipitation of both solids. The measurements were based on looking at the absorption band of the C=O double bond situating around 200–230 nm. The interference stemming from UV-active impurities, most probably Fe(III), made a sound interpretation of these spectra impossible. Therefore, it turned out to be necessary to separate the various components of the system prior to analysis.

Accordingly, HPLC measurements were carried out. The samples included (i) Mg(OH)_2_ filtered after a 1 min reaction time, which was then washed (see Section 2.2); (ii) the gypsum precipitated from the mother liquor, from which the above Mg(OH)_2_ was separated; (iii) the washing fluid used to remove gypsum from the Mg(OH)_2_ (see Section 2.2) and (iv) the mother liquor obtained after gypsum precipitation. The first two solid samples were dissolved in the eluent (0.01 M sulfuric acid). Because of solubility issues, the concentration of the target ion—citrate—approached the detection limit of the HPLC technique, and an accurate concentration determination was not possible. The results, however, strongly suggested that the citrate ions reside in part on the surface of the Mg(OH)_2_ and in part remain in the mother liquor to inhibit the precipitation of gypsum. In the precipitated gypsum and in the washing fluids, no sign of citric acid was observed.

**Figure 10:**
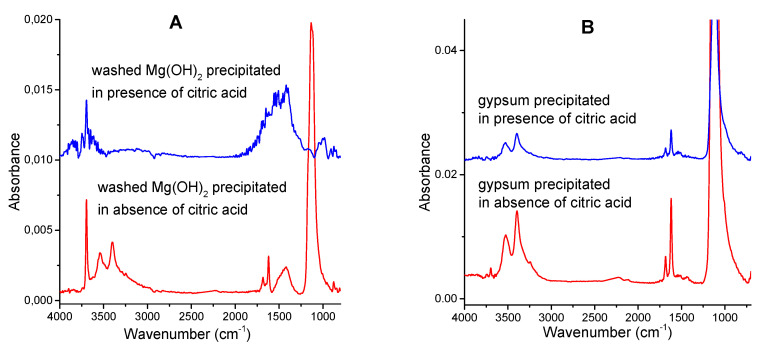
FT-IR spectra of the (**A**): Mg(OH)_2_ and (**B**): CaSO_4_·2H_2_O precipitates obtained from reaction mixtures containing citrate ion (upper spectra) and those in absence of the inhibitor (lower spectra), as described in the text.

To further verify this finding, FT-IR spectroscopy was employed. The FT-IR spectra of solids (i) and (ii) were recorded. To obtain a reference sample, the precipitation reaction was carried out without added citric acid, and the reference solids were obtained and treated as above. During this “uninhibited” reaction, the precipitation of the gypsum is much faster; therefore, it was inevitable that some gypsum remained on the surface of the Mg(OH)_2_ even after washing. The results of these FT-IR measurements are shown in Figure 10.

As it can be seen on both gypsum spectra (Figure 10B), only the characteristic vibration bands of CaSO_4_·2H_2_O are present. The two spectra are practically identical, displaying the typical vibrations of sulfate ions and crystalline water. The bands at the 1100–1200 cm^−1^ region are the signals of ν3(SO_4_), and the broad peaks around 2200 cm^−1^ are combinations of the ν1 and ν2 modes of sulfate. The sharp peaks at 1620 and 1680 cm^−1^ correspond to the ν2 vibrations of crystal water, and the stretching modes of water peak out of the broad band at the region of 3000–3600 cm^−1^ [41]. The spectra of the two (largely) Mg(OH)_2_ samples are, however, very much different. The spectrum of the solid obtained from the reaction without added citric acid is clearly the combination of the spectra of CaSO_4_·2H_2_O and Mg(OH)_2_. Besides the earlier described signs of gypsum, at 3700 cm^−1^, one can see the sharp absorption band of OH stretching of the structural OH groups of Mg(OH)_2_, and also the broad peak in the 1400–1600 cm^−1^ region can be assigned to the water bend vibrations of the adsorbed water [41,42], which is, as noted above, more or less expected. On the other hand, from Figure 8A, the sample taken from the reaction with added citric acid did not contain appreciable amounts of gypsum, and even the peaks of Mg(OH)_2_ are partially masked by some new vibration bands that are not present on the other three spectra. Based on literature data [43,44], the bands in the 1400–2000 cm^−1^ region and above 3500 cm^−1^ correspond to some form of citrate ion, and may well be either magnesium or calcium citrate or some mixed salt of the calcium-magnesium-citrate type [43,44]. This explains the small amounts of calcium, which were detected by the SEM-EDX measurements; citrate is most probably adsorbed on the surface of very fine Mg(OH)_2_ precipitate, and the calcium ions are there to compensate its charge.

The results suggest that Mg(OH)_2_ binds some of the citric acid, which can explain the decreased effectiveness of inhibition compared to the reactions of Na_2_SO_4_ + CaCl_2_ + 2 H_2_O → 2 NaCl + CaSO_4_·2H_2_O. Since this causes a fraction of the additive to be removed from the solution, only the remaining citrate can inhibit the reaction. This must also be considered when working with an increased initial reactant concentration, since a bigger amount of Mg(OH)_2_ will remove more citrate from the solution decreasing the efficiency of inhibition.

Even so, these results are promising for the reusability of the citrate, the precipitated and washed Mg(OH)_2_ containing part of it can be reused in the first step of the neutralization process. The filtrate containing the rest can be potentially used to make up the magnesium oxide in a slurry and also for the first neutralization step.

## 3. Conclusions

During our work, an attempt was made to separate the precipitation of Mg(OH)_2_ and CaSO_4_·2H_2_O in situ by using citrate ions as an additive in the MgSO_4_ + Ca(OH)_2_ + 2H_2_O → Mg(OH)_2_ + CaSO_4_·2H_2_O reaction. The effects of citric acid concentration, the initial reactant concentration and the reaction temperature were investigated. The reactions were monitored by in situ conductometric measurements. The conductometric observations were backed up by ICP-OES measurements. In the target reaction, the Mg(OH)_2_ precipitated instantaneously after the two components were mixed. The precipitation of gypsum was found to be significantly slower, even in the absence of any inhibitor. Adding citric acid to the system exerted no effect on the rate of precipitation of Mg(OH)_2_, but increased significantly the induction time of the gypsum precipitation. Increasing the initial reactant concentration while keeping the additive ratio constant still resulted in an increase in the reaction rate. The increase in the concentration of the added citric acid also increased the induction period notably. With an increasing temperature, the rate of the precipitation reaction was found to increase.

Our results show that with careful control over the reaction conditions, the precipitation of magnesium hydroxide can be separated in time from the precipitation of gypsum using relatively small concentrations of citric acid as an additive. The Mg content of the starting solution can be recovered as relatively pure Mg(OH)_2_ after washing off the inevitable gypsum crystals from its surface. The inhibition effect of citrate is due to the interaction of the anion with the crystal face of the gypsum which is the fastest growing one in the absence of the inhibitor. This effect results in severe morphological changes in the precipitate. The separation of the two precipitation processes can be exploited in different areas—e.g., in the processing of acidic wastewater and mine drainages via neutralization. The observation that the citrate ions partially precipitate on the surface of the Mg(OH)_2_ is even more helpful, since the product as well as the additive can be reused in the neutralization process reducing the cost of the process.

## 4. Materials and Methods

### 4.1. Materials

Magnesium sulfate heptahydrate, 99.7% assay (MgSO_4_⋅7H_2_O), citric acid, 99.9% assay (C_6_H_8_O_7_), were used in this study and are both products of VWR. Other materials used include: calcium oxide, quicklime made from the limestone of a natural source (CaO), provided to us by Lhoist-Minerals lime producer and distilled water.

### 4.2. Preparation of Milk of Lime (MoL)

To get MoL with approximately 20 *w*/*w*% solid content, 155 g of quicklime was suspended in 845 g of distilled water in a cylindrical glass reactor (1-L capacity) with vigorous agitation using a shaft stirrer with a screw propeller PTFE shaft (agitation speed was 800 rpm). The quicklime was added to the distilled water in small portions to avoid quick warming. The system was stirred for 2 h, then the insoluble grids were removed with a sieve with 300 µm hole-diameter. The exact solid content was determined by measuring the weight loss of a heated sample—100 g MoL was dried at 120 °C for 12 h. To ease the operation, the MoL was diluted to ca. 5 *w*/*w*% solid content and the active Ca(OH)_2_ content was determined by titration with HCl using phenolphthalein indicator.

The MoL used for the reactions was always freshly prepared—no samples older than 5 days were used, as the aggregation, sedimentation and reaction with airborne CO_2_ change the properties of MoL significantly after this time period.

### 4.3. Performing the MgSO_4_ + Ca(OH)_2_ + 2H_2_O→ Mg(OH)_2_ + CaSO_4_·2H_2_O Reactions

The reactions were carried out in a cylindrical glass reactor also used for the preparation of the MoL. The agitating system was also the same. The agitation speed chosen was 700 rpm to ensure the quick homogenization of the reaction slurry. The reactions were always carried out in 1 L volume. The magnesium sulfate and the citric acid were made up in distilled water and agitated in the reactor; then, MoL—containing stochiometric amount of Ca(OH)_2_—was added to the system to commence the reaction. MoL with 5 *w*/*w*% solid content was used, the volume of the required amount determined the volume of distilled water in which the MgSO_4_ and citric acid was dissolved. The reactions were followed in situ by conductometric measurements, and the pH and temperature of the reaction were also monitored. The repeatability of the reactions were tested and besides the effect of temperature, the results show that with good control over the experimental conditions the runs of the reactions are well reproducible, as shown in the Supplementary Materials, Appendix B.

To analyze the solids, samples were withdrawn at given times from the slurry, and they were filtered with using vacuum filtration as quickly as possible (note that filtration time is strongly dependent on the volume of the taken sample). The Mg(OH)_2_ samples were washed with distilled water; four times the volume of the sample taken was usually sufficient to wash out gypsum from the samples.

### 4.4. Experimental Methods

Conductometric measurements were carried out using a Jenway 3540 pH and conductivity meter (Keison. Products, Essex, UK) and a Jenway 027,013 conductivity cell. The temperature of the reactions was also followed by the built-in sensor of this conductivity cell.

The pH of the reaction mixtures was measured with a Sentix 62 pH electrode (WTW, Weilheim, Germany), calibrated to 5 points, namely pH 2, 4, 7, 10, 11.5.

For the ICP-OES measurements, samples were taken from the slurry at given times using a syringe, then were quickly filtered with a syringe filter (0.45 µm). The filtrate was diluted one-hundred-fold in 2% nitric acid to avoid further precipitation. The measurements were carried out using a Thermo Scientific iCAP 7400 ICP-OES DUO spectrometer (Waltham, MA, USA).

The diffractograms of the solids were obtained using a Rigaku MiniFlex II type Röntgen diffractometer (Tokyo, Japan), in the 2θ = 4°–60° range with 2°/min scanning speed, using CuKα (λ =  1.5418 Å) radiation.

The morphologies of the precipitated solids were studied by scanning electron microscopy, using a Hitachi S-4700 scanning electron microscope (Tokyo, Japan). The elemental mapping was also possible using the coupled Röntec QX2 spectrometer (Berlin, Germany) equipped with Be window.

The eluent for the HPLC measurements was 0.01 M sulfuric acid; therefore, two-fold diluted fluid samples were prepared. To make samples from the solid samples with maximum citrate concentration, they were dissolved in 0.01 M sulfuric acid to obtain saturated solution, then the remaining solids were filtered off and the fluid sample was diluted two-fold. Each dilution was needed to avoid solubility issues during the measurements. For the measurements, Agilent 1100 series HPLC (Santa Clara, CA, USA) was used with a Grom-Resin ZH type column.

IR measurements were carried out using a JASCO FT/IR-4700 spectrophotometer (Kyoto, Japan) with 4 cm^−1^ resolution accumulating 256 scans in the 4000–650 cm^−1^ wavenumber range. The spectrometer was equipped with a ZnSe ATR accessory and a DTGS detector.

## Figures and Tables

**Table 1:** Correlation between the reaction mixture’s temperature (±0.5 °C) and the rate of the gypsum precipitation reaction.

Temperature (°C)	Estimated Induction Time (min)	Half-Reaction Time (min)
20	10.3	22.8
22	9.1	21.5
24	6.8	17.5
26	5.8	15.7

**Table 2:** Summary of gypsum content in the washed precipitated solids taken at given times in the MgSO_4_ + Ca(OH)_2_ → Mg(OH)_2_ + CaSO_4_·2H_2_O reaction carried out with different initial reactant concentrations and keeping the reactant–additive ratio constant (molar ratio of Ca:Mg:Citric acid = 0.1:0.1:0.0015).

Sample Taken after	Initial Reactant Concentration		
	0.1 M	0.125 M	0.15 M
1 min	No gypsum present	No gypsum present	Gypsum present
5 min	-	Small amount of gypsum present	Gypsum present
10 min	No gypsum present	Gypsum present	Gypsum present
20 min	Gypsum present	Gypsum present	-

## Supplementary Materials

The following are available online, Table S1: The effect of additives on gypsum precipitation in the reaction of Na_2_SO_4_ + CaCl_2_ + 2 H_2_O → 2 NaCl + CaSO_4_·2H_2_O with 0.2 M initial reactant concentrations; Table S2: The effect of some additives on gypsum precipitation in the reaction of MgSO_4_ + Ca(OH)_2_ + 2 H_2_O → Mg(OH)_2_ + CaSO_4_·2H_2_O with 0.2 M initial reactant concentrations; Figure S1: Variation of conductivity during three parallel reactions of MgSO_4_ + Ca(OH)_2_ + 2 H_2_O → Mg(OH)_2_ + CaSO_4_·2H_2_O with 0.1 initial reactant concentration and in presence of 1.5 mM citric acid, at 22 °C.
