# Supplementary material for: Differential Precipitation of Mg(OH)2 from CaSO4·2H2O Using Citrate as Inhibitor—A Promising Concept for Reagent Recovery from MgSO4 Waste Streams
Source: Molecules. 2020 Oct 29;25(21):5012. doi: 10.3390/molecules25215012 (PMC7662945; doi:10.3390/molecules25215012)
Supplement: Supplementary file 1 [file molecules-25-05012-s001.pdf]

molecules

*Electronic supporting information*

# Differential precipitation of $Mg(OH)_2$ from $CaSO_4 \cdot 2H_2O$ using citrate as inhibitor – a promising concept for reagent recovery from $MgSO_4$ waste streams

**Szilveszter Ziegenheim [1,4], Márton Szabados [2,4], Zoltán Kónya [3,5], Ákos Kukovecz [3], István Pálinkó [2,4] and Pál Sipos [1,4,*]**

[1] Department of Inorganic and Analytical Chemistry, University of Szeged; Dóm tér 7., Szeged, H-6720 Hungary

[2] Department of Organic Chemistry, University of Szeged; Dóm tér 8., Szeged, H-6720 Hungary

[3] Department of Applied and Environmental Chemistry, University of Szeged; Rerrich Béla tér 1., Szeged, H-6720 Hungary

[4] Material and Solution Structure Research Group, Institute of Chemistry, University of Szeged, Aradi vértanúk tere 1, Szeged, H-6720 Hungary

[5] MTA-SZTE Reaction Kinetics and Surface Chemistry Research Group, Rerrich B. tér 1, Szeged, H-6720 Hungary

* Correspondence: sipos@chem.u-szeged.hu; Tel.: (optional) +36-62-54-4045 (F.L.)

*Molecules* **2019**, *24*, 5012 doi:10.3390/molecules25215012 

## Appendix A

During our scouting experiments a number of additives were tested in systems containing no $Mg^{2+}$ in the reaction of $Na_2SO_4 + CaCl_2 + 2\ H_2O \rightarrow 2\ NaCl + CaSO_4{\cdot}2H_2O$ with 0.2 M initial reactant concentrations at pH ≈ 7. The amount of additives used was calculated considering economical motives, and their effectiveness was compared with the half reaction time which was determined as described in chapter 2.1 of the main article. The results are summarized in Table S1.

**Table S1.** The effect of additives on gypsum precipitation in the reaction of $Na_2SO_4 + CaCl_2 + 2\ H_2O \rightarrow 2\ NaCl + CaSO_4{\cdot}2H_2O$ with 0.2 M initial reactant concentrations

| **Additive** | **Applied additive concentration (mmol/L)** | **Half-reaction time - i (min)** | **Standard error of i (min)** | **Remark** |
|---|---|---|---|---|
| without additive | - | 0.75 | 0.018 | |
| trisodiumcitrate(dihydrate) | 3.0 | **7.61** | 0.16 | |
| Na-gluconate | 4.0 | 0.87 | 0.016 | |
| sucrose | 1.5 | 0.72 | 0.013 | |
| glycerol | 12.0 | 0.64 | 0.012 | |
| ethylene-glycol | 18.0 | 0.80 | 0.017 | |
| polyethylene glycol (PEG 400) | 1.4 | 0.68 | 0.013 | |
| Na-polyacrylate (MW *ca.* 1200) | 3.0 | - | - | Exceptionally long reaction time |
| K-Na-tartarate | 2.0 | 0.98 | 0.014 | |
| SDS | 1.0 | 1.20 | 0.017 | Foaming |
| diethylenetriamine penta(methylene phosphonic acid) Na salt, DTPMP | 1.2 | - | - | No changes in conductivity for six hours, seemingly colloid formed |

In these reactions the carboxylate salts were used to achieve near neutral pH. While sodium citrate showed moderate effect, sodium polyacrylate and DTPMP seemed to work remarkably well, increasing the induction period up to six hours. Therefore, these three additives were tested in the target reaction of $MgSO_4 + Ca(OH)_2 + 2\ H_2O \rightarrow Mg(OH)_2 + CaSO_4{\cdot}2H_2O$ with 0.2 M initial reactant concentration, using milk of lime as $Ca(OH)_2$ source. The results were compared similarly as before, and are shown on Table S2.

The effectiveness of both sodium polyacrylate and DTPMP dropped drastically, under these conditions they were less effective than sodium citrate, which lost only part of its effect in this system. This can be explained according to our results found later. Probably the polyacrylate and DTPMP were also coordinating to the surface of the precipitating $Mg(OH)_2$, however this coordination was

much stronger than the coordination of citrate, and there was not enough additive left in the mother liquor to effectively inhibit the precipitation of gypsum.

The results suggested that citrate could be effectively used as an inhibitor of gypsum precipitation even in the presence of $Mg(OH)_2$, therefore it was studied in more detail.



**Table S2.** The effect of some additives on gypsum precipitation in the reaction of $MgSO_4 + Ca(OH)_2 + 2\ H_2O \rightarrow \underline{Mg(OH)_2} + \underline{CaSO_4{\cdot}2H_2O}$ with 0.2 M initial reactant concentrations

| **Additive** | **Applied concentration (mmol/L)** | **Halfreaction time - i (min)** | **Standard error of i (min)** |
|---|---|---|---|
| without additive | - | 1.2 | 0.007 |
| trisodiumcitrate(dihydrate) | 3.0 | 3.89 | 0.020 |
| Na-polyacrylate (MW *ca.* 1200) | 3.0 | 2.50 | 0.014 |
| diethylenetriamine penta(methylene phosphonic acid) Na-salt DTPMP | 1.2 | 1.84 | 0.011 |

**Appendix B**

With strict control over the reaction conditions, the repeatability of the reactions was found to be satisfactory, however, as the initial temperature of the reaction mixture was not controlled in our reactions, temperature changes in the environment yielded the most significant differences in the kinetics of the reactions. On Figure S1 the variation of conductivity is presented during three parallel reactions of $MgSO_4 + Ca(OH)_2 + 2\ H_2O \rightarrow \underline{Mg(OH)_2} + \underline{CaSO_4{\cdot}2H_2O}$, where the initial temperature of the reaction mixture was 22.0± 0.5°C $^{o}$C

The initial phase of all three reactions are similar, the induction period variation is about 0.5 minutes while the (presumably more accurate) half-reaction time varies within 0.3 minutes between the parallel runs.

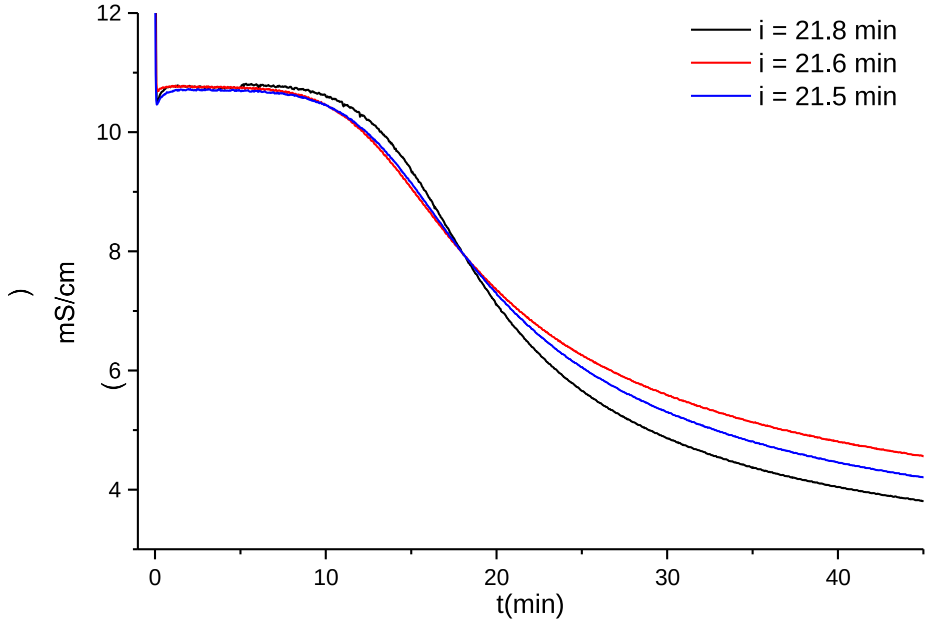


**Figure S1.** Variation of conductivity during three parallel reactions of $MgSO_4 + Ca(OH)_2 + 2\ H_2O \rightarrow$ $\underline{Mg(OH)_2}$ + $\underline{CaSO_4 \cdot 2H_2O}$ with 0.1 initial reactant concentration and in presence of 1.5 mM citric acid, at 22 °C.
